# Impact of vertical facial patterns and oral habits on microbial colonization in children and adolescents: a cross-sectional study

**DOI:** 10.1186/s12887-026-06732-7

**Published:** 2026-03-11

**Authors:** Saliha Akçay Köprücü, Saadettin Kayıpmaz, İlknur Tosun, Ömer Said Sezgin

**Affiliations:** 1 Department of Oral and Maxillofacial Radiology, Faculty of Dentistry, Karadeniz Technical University, Trabzon, Turkey; 2 Department of Medical Microbiology, Faculty of Medicine, Karadeniz Technical University, Trabzon, Turkey

**Keywords:** *Candida*, Gram-positive bacteria, Vertical dimension, Child, Adolescent

## Abstract

**Background:**

The oral commissures represent a unique anatomical interface where intra- and extra-oral environments converge. This study aimed to investigate the association between vertical facial patterns and microbial colonization at the oral commissures in children and adolescents.

**Methods:**

A total of 150 healthy participants (mean age 13.4 ± 2.7 years; range: 8–18 years) were equally divided into low-, norm-, and high-angle groups according to their cephalometric GoGn/SN values. Swab samples from both oral commissures were cultured for *Candida* species and Gram-positive bacteria. Commissural wetness, intraoral pH, and oral habits were recorded. Group differences were analysed using chi-square and one-way ANOVA tests (α = 0.05).

**Results:**

Vertical facial morphology showed a significant association with bacterial colonization findings (*p* = 0.001). Gram-positive bacilli were absent in low-angle but frequent in norm- and high-angle individuals. *Candida* carriage (12.0%) showed no significant association with facial pattern (*p* = 0.103). Mouth breathing was significantly more prevalent in high-angle participants (*p* = 0.038), while lip-licking habits showed no intergroup difference.

**Conclusion:**

Increased vertical facial dimension was associated with bacterial, but not fungal, findings at the oral commissures. Craniofacial morphology may therefore be related to local microbial findings and could be considered a potential modifying factor in paediatric patients presenting with recurrent angular lesions.

**Supplementary Information:**

The online version contains supplementary material available at 10.1186/s12887-026-06732-7.

## Introduction

Vertical facial morphology may influence several local physiological and microbial conditions. Individuals with increased vertical dimensions often exhibit altered perioral muscle tone, lip competence, and salivary flow, which can modify surface moisture and pH at the mouth corners [1–4].

The oral commissures, located at the intersection of intra- and extra-oral regions, represent a dynamic microenvironment affected by facial structure, saliva flow, and oral habits. Imbalances in this area have been associated with microbial accumulation and inflammatory changes [5–7].

Among the microorganisms inhabiting the commissural region, *Candida* species and Gram-positive bacteria are the most frequently detected. *Candida albicans* is a commensal yeast that can become opportunistic under local or systemic disturbances, whereas Gram-positive cocci and bacilli are part of the resident flora that may increase under altered moisture or pH [6, 8, 9].

Previous studies have mostly focused on elderly or denture-wearing populations, where loss of vertical dimension was considered a risk factor for angular cheilitis [6, 9–11]. However, little is known about whether vertical facial patterns in children and adolescents—who exhibit active growth and no prosthetic alterations—affect local microbial colonization at the oral commissures.

In children and adolescents, additional local factors such as mouth breathing and lip-licking habits may play an important role in shaping the microbial findings of the perioral region. Indeed, recent studies have shown that mouth breathing not only is associated with skeletal and facial vertical changes (for example, increased mandibular plane angles) but also corresponds to distinct alterations in oral microbial communities, including enrichments of opportunistic bacterial species and changes in salivary protein composition [12, 13]. These oral habits are also known to be more prevalent in certain vertical facial patterns, such as high-angle skeletal configurations, and may therefore be considered potential modifying factors in relation to microbial findings. Despite these potential links, evidence regarding the interaction between vertical facial patterns, oral habits, and oral microbial colonization in children and adolescents remains scarce.

Therefore, the aim of this study was to evaluate the relationship between vertical facial morphology, oral habits, and microbial colonization at the oral commissures in children and adolescents. We hypothesized that distinct vertical growth patterns could be associated with specific microbial profiles and that oral habits may influence this relationship. To our knowledge, this is the first study to highlight the oral commissures as a clinically relevant site reflecting both functional and microbial interactions in growing individuals.

## Materials and methods

### Ethical approval

This study was reviewed and approved by the Scientific Research Ethics Committee of Karadeniz Technical University, Faculty of Dentistry (Approval No: 2015/89). All procedures were conducted in accordance with the Declaration of Helsinki, and written informed consent was obtained from all participants and their parents or legal guardians.

### Study design and sample selection

This cross-sectional study included 150 children and adolescents (66 males, 84 females; mean age = 13.4 years; range 8–18 years) who were referred for orthodontic evaluation prior to treatment at the Department of Oral and Maxillofacial Radiology, Faculty of Dentistry, Karadeniz Technical University. Participants were equally distributed into low-, norm-, and high-angle groups based on cephalometric criteria to allow balanced comparisons across vertical facial patterns. The study was conducted in accordance with the STROBE checklist for cross-sectional studies [14].

Eligibility was restricted to systemically healthy individuals with no history of tobacco or alcohol use, no previous orthodontic treatment, and no craniofacial anomalies. Participants who had received systemic antibiotic therapy within the four weeks prior to sampling were excluded to minimize potential alterations in oral microbial composition. All clinical and radiological assessments were performed by a single calibrated examiner (S.A.K.).

Caries-free status was defined as the absence of clinically detectable cavitated carious lesions and radiographic evidence of dentinal caries at the time of examination. Individuals with active or untreated carious lesions, gingivitis, periodontitis, mucosal pathology, or other inflammatory oral conditions were excluded. Any incipient (non-cavitated) carious lesions, if previously present, had been treated and were not active at the time of inclusion.

### Assessment of vertical facial dimension

Lateral cephalometric radiographs were obtained with a Kodak 9000 C Dental System (Carestream Health, Rochester, NY, USA) using 80 kVp, 10 mA, and 0.5 s exposure, with a 24 × 30 cm field of view and a magnification factor of 1.15. All cephalometric analyses were performed by a single calibrated examiner (S.A.K.) with over 10 years of experience in dentomaxillofacial radiology. The radiographs were digitized at a resolution of 300 dpi, and measurements were performed using Dolphin Imaging Software (v. 11.7, Dolphin Imaging and Management Solutions, Chatsworth, CA, USA). Vertical facial pattern was defined by the GoGn/SN angle and classified as low-angle (< 26°), norm-angle (26–36°), or high-angle (> 36°). To evaluate intra-observer reliability, 30 lateral cephalometric radiographs were randomly selected and re-measured by the same examiner one week after the initial analysis. Intra-observer agreement for the GoGn/SN angle was assessed using the Intraclass Correlation Coefficient (ICC) with a 95% confidence interval. The ICC value for the GoGn/SN angle was 0.97, indicating excellent intra-observer reliability.

### Oral habits assessment

Breathing pattern and lip-licking habit information were obtained by self-report from children and their parents and recorded as either present or absent.

### Oral pH measurement

Intraoral pH was measured using MColorpHast indicator strips (Merck, Darmstadt, Germany). Strips were placed intraorally for 2 min, then compared with the manufacturer’s color chart.

### Angular wetness measurement

Angular wetness was assessed with modified Schirmer strips folded in an L shape. The shorter arm (5 mm) was positioned at the mouth corner for 3 min, and the wetted length (mm) recorded as the wetness value.

### Collection and culturing of swab samples

All samples were collected between 9:00 and 11:00 AM to minimize diurnal variations. Using sterile cotton swabs, both commissures were sampled by gentle contact for ≥ 5 s. Each swab was transferred to Stuart transport medium (FIRATMED, Türkiye) and immediately inoculated on Sabouraud dextrose agar (SDA) in duplicate Petri dishes, incubated 30–35 °C for 7 days. Colonies were microscopically examined; yeast-like forms underwent the germ-tube test (positive = *C. albicans*), while negative samples were classified as non-albicans *Candida* using API 20 C AUX (bioMérieux, France).

### Statistical analysis

All analyses were performed in SPSS v26.0 (IBM Corp., Armonk, NY, USA). Sample adequacy (*N* = 150; *n* = 50 per group) was verified using G*Power 3.1.9.7 for one-way ANOVA (effect size 0.30, α = 0.05, power = 0.91).

Normality of continuous variables (age, wetness, pH) was verified with Kolmogorov–Smirnov and |skewness|, |kurtosis| < 1.5; hence parametric tests were applied. Means ± SD and frequencies (%) summarized data.

Group differences for continuous variables were tested with one-way ANOVA and Duncan post-hoc comparisons. Categorical associations (e.g., vertical pattern vs. microbial type, oral habit) used Pearson χ² and Fisher’s exact test (where expected frequencies < 5). Correlations among continuous variables used Pearson r. Two-tailed *p* < 0.05 was considered significant.

## Results

### Microbiological findings

Microbiological analysis showed that Gram-positive cocci were the most prevalent isolates, found in 84 of 150 participants (56.0%). Other findings included no microbial growth in 23 (15.3%), *Candida* species in 18 (12.0%), Gram-positive bacilli in 18 (12.0%), and combined Gram-positive cocci + bacilli in 7 (4.7%) (Table 1). Overall, Gram-positive cocci represented the dominant flora at the oral commissures of healthy children and adolescents.

**Table 1:** Distribution of demographic, microbiological, and oral environmental variables in the study population

Variable	Category	*N*	%
Vertical Facial Pattern	Low-angle	50	33.3%
	Norm-angle	50	33.3%
	High-angle	50	33.3%
Microbiological Findings	No growth	23	15.3%
	*Candida* spp.	18	12.0%
	Gram-positive cocci	84	56.0%
	Gram-positive bacilli	18	12.0%
	Gram-positive cocci + bacilli	7	4.7%
Lip-licking habit	Absent	131	87.3%
	Present	19	12.7%
Mouth breathing	Absent	117	78.0%
	Present	33	22.0%

		Mean	Std. Dev.
Age (years)		13.41	2.71
Oral commissural wetness (mm)		9.44	5.00
Intraoral pH		7.30	0.35
Vertical dimension degree (°)		31.25	8.33

**Fig. 1:**
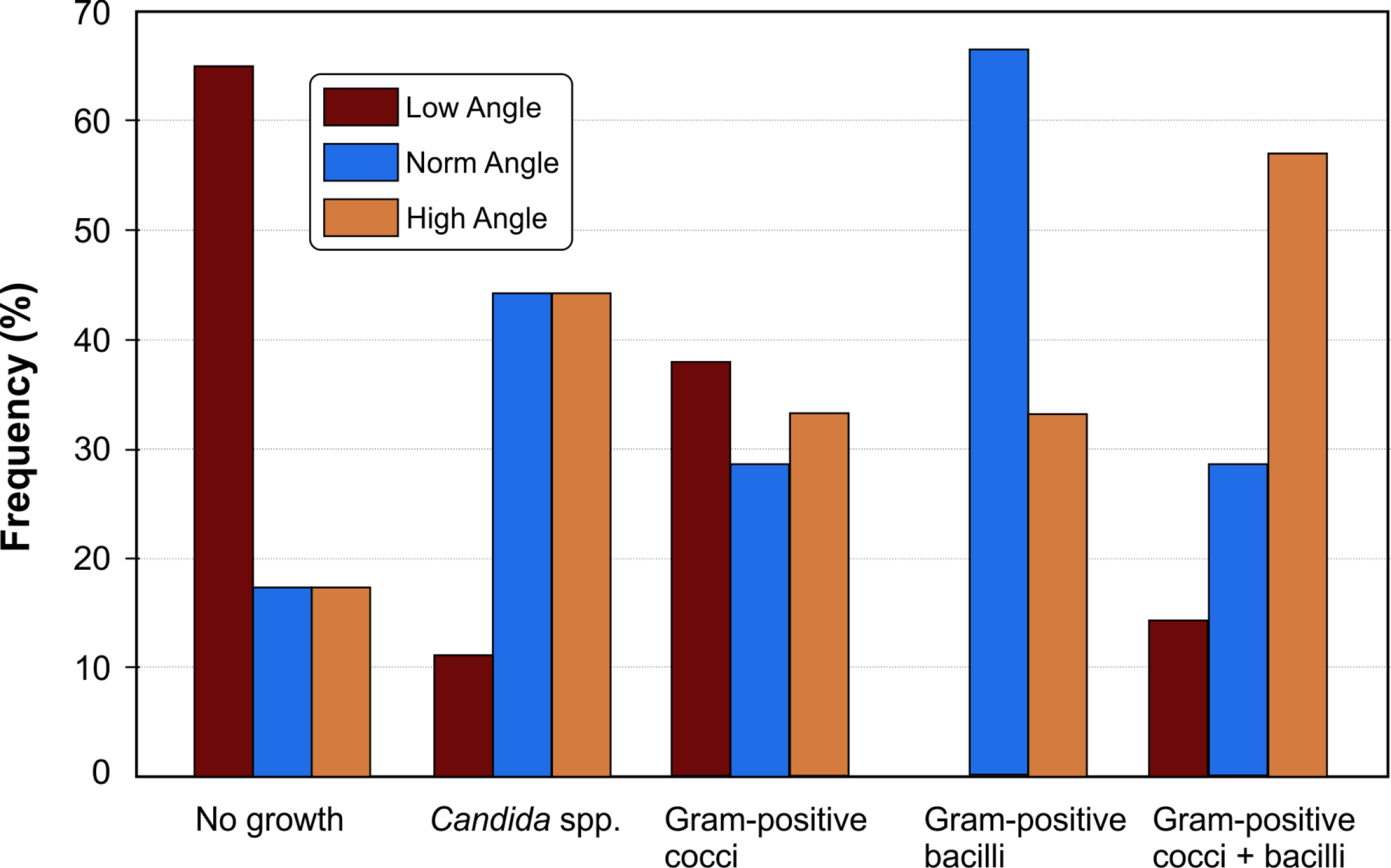
Prevalence of microbial isolates at the oral commissures by vertical facial dimension

A significant association was observed between vertical facial morphology and microbial outcomes (*p* = 0.001) (Table 2). Post-hoc analysis indicated that the low-angle group had a higher rate of no microbial growth (65.2%) compared with other groups, whereas all Gram-positive bacilli isolates occurred in norm-angle (66.7%) and high-angle (33.3%) individuals (Fig. 1).

**Table 2:** Relationship between vertical facial morphology. microbial colonization. and oral habits

		Low Angle (*n* = 50)		Norm Angle (*n* = 50)		High Angle (*n* = 50)			
		N	%	N	%	N	%	*χ* ^*2*^	*p.
Microbiological findings	No growth	15	65.2%	4	17.4%	4	17.4%	*29.665*	***0.001***
	*Candida* spp	2	11.1%	8	44.4%	8	44.4%		
	Gram-positive cocci	32	38.1%	24	28.6%	28	33.3%		
	Gram-positive bacilli	0	0.0%	12	66.7%	6	33.3%		
	Gram-positive cocci + bacilli	1	14.3%	2	28.6%	4	57.1%		
Lip-licking habit	Absent	41	31.3%	48	36.6%	42	32.1%	*5.183*	*0.075*
	Present	9	47.4%	2	10.5%	8	42.1%		
Mouth breathing	Absent	43	36.8%	41	35.0%	33	28.2%	*6.527*	***0.038***
	Present	7	21.2%	9	27.3%	17	51.5%		

Bold *p*-values indicate statistically significant results (*p* ≤ 0.05)

*Statistical significance was determined based on chi-square test results

One-way ANOVA revealed significant differences in vertical dimension degrees across microbial categories (*p* = 0.005) (Table 3). Participants with *Candida* (35.9 ± 6.8), Gram-positive bacilli (33.2 ± 3.7), Gram-positive cocci (30.8 ± 9.3), or cocci + bacilli (34.7 ± 5.8) had higher GoGn/SN angles than those with no microbial growth (26.8 ± 6.6).

**Table 3:** Comparison of physiological parameters according to microbiological findings

	No growth		Candida spp.		Gram-positive cocci		Gram-positive bacilli		Gram-positive cocci + bacilli			
	Mean	SD	Mean	SD	Mean	SD	Mean	SD	Mean	SD	F	*p.
Age	12.78	2.84	13.11	2.87	13.92	2.67	12.50	2.38	12.43	2.37	1.881	*0.117*
Oral commissural wetness (mm)	9.72	5.12	8.53	3.60	9.30	4.76	10.28	7.03	10.36	5.30	0.362	*0.835*
Intraoral pH	7.33	0.42	7.33	0.45	7.29	0.32	7.25	0.26	7.43	0.35	0.435	*0.783*
Vertical dimension degree (°)	26.83^b^	6.55	35.86^a^	6.75	30.79^a^	9.31	33.15^a^	3.69	34.71^a^	5.83	3.869	***0.005***

Bold *p*-values indicate statistically significant results (*p* ≤ 0.05)

*Statistical significance was determined using one-way ANOVA; different superscript letters (a, b) indicate statistically significant intergroup differences according to the Duncan post-hoc test

*Candida* carriage showed no significant association with vertical facial pattern (*p* = 0.103). Similarly, no significant differences were found for cocci or cocci + bacilli combinations (all *p* > 0.05). In addition, no significant sex-related differences were observed in any of the microbiological outcomes (all *p* > 0.05).

### Angular wetness, oral pH, and age distribution

Mean angular wetness was 9.44 ± 5.0 mm and mean intraoral pH = 7.3 ± 0.3 (Table 1). Neither angular wetness nor intraoral pH differed significantly among the low-, norm-, and high-angle groups (all *p* > 0.05) (Table 4). Correlation analysis indicated that age correlated negatively with wetness (*r* = − 0.369, *p* = 0.001), while wetness (*r* = 0.171, *p* = 0.036) and pH (*r* = 0.183, *p* = 0.025) correlated positively with vertical dimension degrees (Table 5). Thus, higher vertical dimensions were associated with slightly higher moisture and pH, whereas increasing age reduced wetness. In addition, there were no significant sex-related differences in the physiological parameters, including pH and angular wetness (all *p* > 0.05).

**Table 4:** Comparison of age, oral commissural wetness, and intraoral pH according vertical facial pattern

	Vertical Dimension							
	Low Angle		Norm Angle		High Angle			
	Mean	SD	Mean	SD	Mean	SD	F	*p.
Age	13.34	2.91	12.92	2.81	13.96	2.32	*1.889*	*0.155*
Oral commissural wetness (mm)	8.95	4.95	8.88	4.20	10.49	5.67	*1.670*	*0.192*
Intraoral pH	7.24	0.34	7.32	0.32	7.34	0.38	*1.161*	*0.316*

*Statistical significance was determined using one-way ANOVA

**Table 5:** Pearson correlation coefficients among age, oral wetness, intraoral pH and vertical dimension degree

		Age	Wetness (mm)	Intraoral pH	Vertical dimension degree (°)
Age	r	1			
	p.				
Wetness (mm)	r	− 0.369**	1		
	p.	0.001			
Intraoral pH	r	0.066	0.108	1	
	p.	0.425	0.189		
Vertical dimension degree (°)	r	0.064	0.171*	0.183*	1
	p.	0.434	0.036	0.025	

*r: Pearson correlation coefficients; *p* < 0.05

### Mouth breathing

Mouth breathing was reported by 22.0% (*n* = 33). Its association with vertical morphology was significant (*p* = 0.038) (Table 2). The habit was most frequent in high-angle participants (51.5%) versus norm-angle (27.3%) and low-angle (21.2%) groups, suggesting that increased vertical dimension relates to greater mouth-breathing prevalence.

Although the association between mouth breathing and microbiological category did not reach significance (*p* = 0.078), descriptive data showed that Gram-positive cocci were most common among mouth breathers (57.6%), followed by *Candida* (21.2%).

### Lip-licking

Lip-licking was reported in 12.7% (*n* = 19). No significant association was found between vertical morphology and lip-licking (*p* = 0.075) (Table 2). Gram-positive cocci predominated among these individuals (68.4%), but the relationship was not statistically significant (*p* = 0.432).

## Discussion

This study demonstrates that vertical skeletal morphology is significantly associated with bacterial—but not fungal—colonization at the oral commissures in children and adolescents. The absence of Gram-positive bacilli in low-angle individuals, contrasted with their presence in norm- and high-angle groups, indicates that craniofacial structure may be an important contributing factor to the perioral ecological niche. To our knowledge, this association has not previously been documented in paediatric populations.

The age range of 8–18 years was deliberately selected to represent late childhood and adolescence. Current evidence indicates that the most substantial age-related changes in the oral microbiome occur during infancy and early childhood, during which microbial communities undergo maturation and establishment [15]. After this developmental phase, the oral microbiota in systemically healthy individuals is considered relatively stable, with variations more strongly influenced by local environmental and host-related factors than by chronological age alone [1, 2]. In addition, population-based data suggest that salivary bacterial composition is more strongly associated with oral ecological parameters than with age per se in healthy individuals [16]. Furthermore, mean age did not differ significantly among the vertical facial pattern groups (Table 4), indicating that age distribution was comparable across groups and was therefore unlikely to substantially influence the observed associations. Therefore, the selected age range and balanced group distribution were considered appropriate for evaluating morphology-related differences while minimizing potential age-related confounding.

The prevalence of *Candida* carriage (12.0%) in this cohort is lower than the 20–40% range commonly reported for children [17, 18]. This difference likely reflects the localized sampling site (oral commissures only) and the inclusion of caries-free, systemically healthy participants. Prior studies have used pooled intraoral samples from multiple sites, resulting in higher isolation rates. Importantly, colonization does not imply infection; *Candida* species often exist as commensals [19].

In contrast, Gram-positive bacilli were more frequently observed in individuals with increased vertical dimension. These organisms were found exclusively in norm- and high-angle individuals, supporting the idea that skeletal morphology may be related to differences in the local microbial environment. Such a relationship extends previous research that largely emphasized fungal species by highlighting how anatomical structure may condition bacterial colonization patterns [20].

Recent evidence suggests that mouth breathing may influence the composition of the oral microbiota. Marincak Vrankova et al. reported a higher relative abundance of *Solobacterium* in mouth-breathing children compared with nasal breathers, indicating a potential microbial shift associated with altered breathing patterns [21]. In our study, however, no significant microbial differences were detected between breathing types. The increased occurrence of Gram-positive bacilli in the high-angle group might indirectly reflect a higher prevalence of mouth breathing within this craniofacial pattern. Nonetheless, subtle microbial alterations related to oral respiration may not have been fully captured, as the breathing pattern was determined based on self-reported information rather than objective evaluation.

Previous microbiome studies have shown that the oral cavity harbors hundreds of bacterial species, primarily from the phyla *Firmicutes*, *Bacteroidetes*, *Proteobacteria*, *Actinobacteria*, *Spirochaetes*, and *Fusobacteria *[22]. In healthy individuals, *Streptococcus salivarius* represents the predominant commensal organism on the tongue surface, whereas its reduction and the increased abundance of anaerobes such as *Solobacterium moorei*, *Atopobium parvulum*, and *Fusobacterium periodonticum* have been associated with halitosis and dysbiotic oral conditions [23]. These findings support the concept that changes in the oral environment—such as those potentially occurring in high-angle, mouth-breathing individuals—may favor a shift toward microbial profiles characterized by increased representation of anaerobic and opportunistic microorganisms.

Mouth breathing was more frequent in high-angle individuals, consistent with earlier studies associating this habit with increased mandibular plane angles and maxillary constriction [24, 25]. Although not statistically significant, mouth breathers showed a tendency toward higher bacterial diversity, possibly related to airflow and mucosal dehydration. The present findings underscore the importance of considering airway-related factors in paediatric assessment. However, no statistically significant association was observed between mouth breathing and microbial findings in the present study. These results suggest that, although mouth breathing is related to vertical facial morphology, it does not independently account for the observed differences in commissural microbial findings.

Lip-licking behaviour was not significantly related to vertical morphology but showed mild directional trends. Repetitive lip licking can increase moisture and epithelial irritation, promoting colonization potential [26]. Larger samples may clarify whether such habits indirectly modulate the commissural microbiota.

Neither fungal nor bacterial findings were significantly associated with oral habits, suggesting that structural morphology may be more consistently related to commissural microbial findings than transient behavioural factors. These observations are in line with contemporary reviews describing the paediatric oral microbiome as multifactorial and influenced by host anatomical characteristics [1, 2, 7].

The mechanisms underlying the association between increased vertical facial dimension and bacterial findings at the oral commissures are likely multifactorial and remain incompletely understood. High-angle individuals often exhibit reduced lip seal competence and altered perioral muscle function, which have been suggested to influence the local commissural environment [27]. In addition, the higher prevalence of mouth breathing observed in the high-angle group (51.5%) may be relevant, given its potential effects on mucosal exposure and local immune responses [12]. Biomechanical factors, such as altered tissue tension at the commissures in vertically excessive facial patterns, may also contribute to epithelial barrier function. It should be noted that the clinical wetness measurement used in this study represents a limited surrogate of the commissural microenvironment and may not completely reflect dynamic or qualitative aspects of local moisture. Accordingly, the absence of significant intergroup differences in pH and wetness suggests that these parameters alone do not explain the observed associations. Other unmeasured factors—such as salivary protein composition, local cytokine profiles, or epithelial turnover rates—may therefore play important roles [28]. Further studies employing molecular and immunological approaches are warranted to clarify these potential pathways.

### Limitations and future directions

This study has several limitations that should be considered when interpreting the results. Although equal group sizes were used to ensure statistical balance, this stratification does not reflect the natural prevalence of vertical facial patterns in the general paediatric population, which may limit the generalizability of the findings. Furthermore, culture-based identification restricted taxonomic resolution; molecular techniques such as 16 S rRNA sequencing would provide broader microbial detection and species-level characterization. Additional variables—including nutritional status, salivary flow rate, and vitamin levels—were not evaluated, and single-time measurements of pH and wetness limit causal inference and indicate that the findings reflect culture-detectable microorganisms at the time of sampling rather than persistent colonization.

Future research should adopt multicentre and longitudinal designs integrating molecular bacteriome and mycobiome profiling, objective quantification of oral habits, and airway assessment. Such studies would help clarify the causal pathways linking craniofacial morphology to oral microbial findings and identify potential clinical markers for early preventive intervention.

## Conclusion

In this cross-sectional study of healthy children and adolescents, increased vertical facial dimension was significantly associated with Gram-positive bacilli findings at the oral commissures, independent of *Candida* carriage. These findings suggest that craniofacial morphology may influence local bacterial ecology and warrant consideration in pediatric oral health assessment.

## Supplementary Information

Supplementary Material 1.

## Data Availability

All data generated or analysed during this study are included in the supplementary material files submitted with the manuscript.

## Abbreviations

ANOVA: Analysis of Variance
CFU: Colony-Forming Unit
FOV: Field of View
SPSS: Statistical Package for the Social Sciences
STROBE: Strengthening the Reporting of Observational Studies in Epidemiology
