# Supplementary material for: Impact of vertical facial patterns and oral habits on microbial colonization in children and adolescents: a cross-sectional study
Source: BMC Pediatr. 2026 Mar 11;26:342. doi: 10.1186/s12887-026-06732-7 (PMC13088411; doi:10.1186/s12887-026-06732-7)
Supplement: Supplementary file 1 — Supplementary Material 1. [file 12887_2026_6732_MOESM1_ESM.docx]

**Supplementary Table 1. Raw patient-level data of the study cohort (n = 150).**

This table presents individual-level data from all participants, including vertical dimension, microbiological findings, age, wetness (mm), pH, lip licking, mouth breathing, and GoGn/SN angle (°). Data are provided to ensure transparency and reproducibility of the study findings.

| Case No | Vertical Dimension | Microbiological Result | Age | Wetness (mm) | pH | Lip Licking | Mouth Breathing | GoGn/SN Angle (°) |
| --- | --- | --- | --- | --- | --- | --- | --- | --- |
| 1 | Low Angle | Absent | 18 | 5 | 7 | Absent | Absent | 23,06 |
| 2 | Norm Angle | Absent | 10 | 3 | 7 | Absent | Absent | 27,72 |
| 3 | Low Angle | Gram-positive cocci | 11 | 8 | 7 | Absent | Occasional | 21,5 |
| 4 | Low Angle | Gram-positive cocci | 14 | 7 | 7 | Continuous | Continuous | 19,48 |
| 5 | Low Angle | Gram-positive cocci | 16 | 6 | 7 | Occasional | Occasional | 25,57 |
| 6 | Low Angle | Gram-positive cocci | 17 | 10 | 7,5 | Absent | Absent | 23,43 |
| 7 | Low Angle | Absent | 17 | 6,5 | 7 | Absent | Absent | 24,8 |
| 8 | Low Angle | Gram-positive cocci | 16 | 5 | 7 | Absent | Absent | 18,2 |
| 9 | Low Angle | Gram-positive cocci | 13 | 7 | 7,5 | Absent | Absent | 25,7 |
| 10 | Low Angle | Candida Spp | 10 | 4 | 8 | Absent | Occasional | 25,8 |
| 11 | Low Angle | Absent | 17 | 3 | 7 | Absent | Absent | 22,8 |
| 12 | Low Angle | Gram-positive cocci | 12 | 15 | 8 | Absent | Absent | 23,38 |
| 13 | Low Angle | Gram-positive cocci | 14 | 4 | 7 | Absent | Absent | 25 |
| 14 | Low Angle | Gram-positive cocci | 15 | 17 | 7 | Occasional | Absent | 15,8 |
| 15 | Low Angle | Absent | 15 | 4 | 7 | Occasional | Absent | 24,13 |
| 16 | Low Angle | Gram-positive cocci | 15 | 7,5 | 7 | Absent | Absent | 23,8 |
| 17 | Low Angle | Absent | 13 | 16 | 7 | Absent | Absent | 20,8 |
| 18 | Low Angle | Absent | 15 | 11 | 7 | Absent | Continuous | 19,5 |
| 19 | Low Angle | Absent | 12 | 8 | 8 | Absent | Absent | 23,2 |
| 20 | Low Angle | Gram-positive cocci | 13 | 3 | 7,5 | Absent | Absent | 24,04 |
| 21 | Low Angle | C. Parapsilosis | 15 | 8 | 7 | Absent | Absent | 21,55 |
| 22 | Low Angle | Absent | 9 | 6 | 7 | Absent | Absent | 18,9 |
| 23 | Low Angle | Gram-positive cocci | 14 | 10 | 7,5 | Absent | Absent | 22,9 |
| 24 | Low Angle | Gram-positive cocci | 8 | 14 | 7,5 | Absent | Absent | 19,4 |
| 25 | Low Angle | Gram-positive cocci | 11 | 10 | 7 | Absent | Absent | 23,3 |
| 26 | Low Angle | Gram-positive cocci | 15 | 17 | 7,5 | Occasional | Absent | 23,1 |
| 27 | Low Angle | Gram-positive cocci | 9 | 8 | 7 | Absent | Absent | 21,9 |
| 28 | Low Angle | Absent | 10 | 13 | 8 | Absent | Absent | 19,2 |
| 29 | Low Angle | Absent | 10 | 22 | 7,5 | Absent | Absent | 24,7 |
| 30 | Low Angle | Gram-positive cocci | 10 | 6 | 7 | Absent | Absent | 16,8 |
| 31 | Low Angle | Gram-positive cocci | 16 | 8 | 7 | Absent | Occasional | 20,5 |
| 32 | Low Angle | Absent | 8 | 13 | 7 | Absent | Absent | 25,12 |
| 33 | Low Angle | Gram-positive cocci | 17 | 1,5 | 7 | Absent | Absent | 25,6 |
| 34 | Low Angle | Absent | 12 | 7 | 7,5 | Absent | Absent | 22,26 |
| 35 | Norm Angle | Gram-positive cocci | 9 | 11 | 7,5 | Absent | Continuous | 30,09 |
| 36 | Low Angle | Absent | 8 | 15,5 | 7 | Occasional | Occasional | 24,8 |
| 37 | Low Angle | Gram-positive cocci | 15 | 13 | 7,5 | Absent | Absent | 25,8 |
| 38 | Low Angle | Gram-positive cocci | 15 | 6 | 7 | Absent | Absent | 22,8 |
| 39 | Low Angle | Gram-positive cocci | 12 | 4 | 7,5 | Absent | Absent | 19,5 |
| 40 | Low Angle | Gram-positive cocci | 14 | 12,5 | 7 | Absent | Absent | 9,6 |
| 41 | Norm Angle | Gram-positive cocci | 8 | 16 | 7,5 | Absent | Absent | 32,55 |
| 42 | Norm Angle | C. Albicans | 11 | 8 | 7 | Absent | Absent | 30,76 |
| 43 | Low Angle | Absent | 15 | 6 | 7,5 | Absent | Absent | 22,18 |
| 44 | Low Angle | Gram-positive cocci | 15 | 5 | 7 | Continuous | Absent | 18,9 |
| 45 | Low Angle | Absent | 14 | 10 | 7 | Absent | Absent | 25,25 |
| 46 | Low Angle | Gram-positive cocci | 12 | 6 | 7,5 | Continuous | Absent | 19,6 |
| 47 | Norm Angle | Gram-positive cocci | 16 | 5 | 7 | Absent | Absent | 27,9 |
| 48 | Norm Angle | Gram-positive cocci | 10 | 7 | 7 | Absent | Absent | 33,6 |
| 49 | Low Angle | Gram-positive cocci | 13 | 4 | 7 | Absent | Absent | 24,13 |
| 50 | Norm Angle | Absent | 10 | 10 | 7 | Absent | Absent | 34,7 |
| 51 | Norm Angle | Gram-positive cocci | 8 | 13 | 7 | Absent | Absent | 31,5 |
| 52 | High Angle | Gram-positive cocci | 16 | 7 | 7 | Absent | Continuous | 37,7 |
| 53 | Low Angle | Gram-positive cocci | 9 | 25 | 7 | Absent | Absent | 24,8 |
| 54 | High Angle | Candida spp | 9 | 14 | 8 | Absent | Continuous | 44,2 |
| 55 | Norm Angle | Gram-positive cocci | 15 | 6 | 7,5 | Absent | Absent | 27,2 |
| 56 | High Angle | Gram-positive cocci | 9 | 14 | 7 | Absent | Absent | 40,8 |
| 57 | High Angle | Gram-positive cocci | 13 | 7 | 7 | Continuous | Continuous | 40,4 |
| 58 | High Angle | Gram-positive cocci | 10 | 11 | 7,5 | Absent | Absent | 36,19 |
| 59 | Norm Angle | Mixed bacterial growth | 12 | 15 | 7,5 | Absent | Absent | 32,9 |
| 60 | Norm Angle | Gram-positive cocci | 14 | 14 | 7,5 | Absent | Absent | 35,4 |
| 61 | Norm Angle | C. Albicans | 9 | 7 | 7 | Absent | Absent | 31,5 |
| 62 | Norm Angle | Gram-positive cocci | 13 | 11 | 8 | Absent | Absent | 28,5 |
| 63 | Norm Angle | Gram-positive cocci | 15 | 9 | 7 | Absent | Absent | 27,4 |
| 64 | Low Angle | Absent | 10 | 12 | 7 | Occasional | Absent | 23,8 |
| 65 | Norm Angle | Gram-positive cocci | 15 | 10 | 7 | Absent | Absent | 28 |
| 66 | Norm Angle | Gram-positive cocci | 15 | 5 | 7 | Absent | Continuous | 28,6 |
| 67 | Norm Angle | Gram-positive cocci | 17 | 3 | 7,5 | Occasional | Absent | 32,5 |
| 68 | Norm Angle | Absent | 12 | 7 | 7 | Absent | Absent | 31,9 |
| 69 | High Angle | Gram-positive cocci | 16 | 13 | 8 | Absent | Continuous | 50,1 |
| 70 | High Angle | Gram-positive cocci | 12 | 14 | 7 | Occasional | Continuous | 40,1 |
| 71 | Norm Angle | Gram-positive cocci | 16 | 7 | 7 | Absent | Absent | 28,75 |
| 72 | High Angle | Gram-positive cocci | 12 | 5 | 7 | Absent | Absent | 39,3 |
| 73 | Low Angle | Gram-positive cocci | 18 | 6 | 8 | Absent | Absent | 20,2 |
| 74 | Low Angle | Gram-positive cocci | 18 | 5 | 7,5 | Absent | Absent | 19,2 |
| 75 | Norm Angle | C. Albicans | 8 | 11 | 7,5 | Absent | Absent | 34,3 |
| 76 | High Angle | Gram-positive cocci | 14 | 4 | 8 | Absent | Occasional | 47,2 |
| 77 | Norm Angle | Gram-positive cocci | 16 | 3 | 7,5 | Absent | Absent | 31,4 |
| 78 | High Angle | Gram-positive cocci | 14 | 9 | 7 | Absent | Absent | 37,9 |
| 79 | Norm Angle | Gram-positive cocci | 17 | 10 | 7,5 | Absent | Absent | 27,5 |
| 80 | High Angle | Gram-positive cocci | 17 | 6 | 8 | Continuous | Continuous | 48,7 |
| 81 | Norm Angle | Absent | 11 | 12 | 8 | Absent | Absent | 30,6 |
| 82 | High Angle | Gram-positive cocci | 13 | 14 | 7 | Absent | Absent | 40,9 |
| 83 | Norm Angle | Candida Spp | 17 | 10 | 8 | Absent | Continuous | 31,6 |
| 84 | High Angle | Gram-positive cocci | 16 | 11 | 7 | Absent | Absent | 39,9 |
| 85 | Low Angle | Gram-positive cocci | 18 | 6 | 7,5 | Absent | Absent | 22,8 |
| 86 | Low Angle | Gram-positive cocci | 11 | 10 | 7 | Absent | Absent | 22,9 |
| 87 | Norm Angle | Gram-positive cocci | 17 | 10 | 7,5 | Absent | Absent | 27,2 |
| 88 | Norm Angle | Candida Spp | 17 | 6 | 8 | Occasional | Absent | 33,07 |
| 89 | High Angle | Gram-positive cocci | 16 | 5 | 8 | Occasional | Absent | 37 |
| 90 | Low Angle | Gram-positive cocci | 13 | 11 | 7 | Absent | Absent | 22 |
| 91 | Norm Angle | Gram-positive cocci | 14 | 11 | 7 | Absent | Continuous | 35,01 |
| 92 | Norm Angle | Gram-positive cocci and bacilli | 11 | 5 | 7 | Absent | Occasional | 31,3 |
| 93 | Norm Angle | Candida Spp | 12 | 11 | 7 | Absent | Absent | 31,3 |
| 94 | High Angle | Gram-positive cocci | 11 | 23 | 7,5 | Absent | Continuous | 44,4 |
| 95 | Norm Angle | Gram-positive cocci | 13 | 13 | 7,5 | Absent | Absent | 29,05 |
| 96 | High Angle | Gram-positive cocci | 14 | 10 | 7,5 | Absent | Absent | 47,8 |
| 97 | Norm Angle | Candida Spp | 16 | 4 | 7,5 | Absent | Absent | 34,7 |
| 98 | Norm Angle | Gram-positive cocci | 15 | 13 | 7,5 | Absent | Absent | 28,9 |
| 99 | Norm Angle | Gram-positive cocci | 9 | 10 | 7,5 | Absent | Absent | 33,6 |
| 100 | Norm Angle | Gram-positive cocci | 14 | 15 | 7,5 | Absent | Occasional | 33,54 |
| 101 | Norm Angle | Gram-positive bacilli | 10 | 10 | 7,5 | Absent | Absent | 32,5 |
| 102 | Norm Angle | Gram-positive bacilli | 12 | 18 | 7,5 | Absent | Continuous | 32,3 |
| 103 | Norm Angle | Gram-positive bacilli | 15 | 3 | 7,5 | Absent | Absent | 32,3 |
| 104 | Norm Angle | Gram-positive bacilli | 13 | 3 | 7 | Absent | Absent | 30,5 |
| 105 | Norm Angle | Gram-positive bacilli | 10 | 12 | 7,5 | Absent | Absent | 29,84 |
| 106 | High Angle | Gram-positive bacilli | 14 | 13 | 7 | Absent | Absent | 36,2 |
| 107 | Norm Angle | Gram-positive bacilli | 14 | 1 | 7,5 | Absent | Absent | 28,19 |
| 108 | Norm Angle | Gram-positive cocci | 12 | 9 | 7,5 | Absent | Absent | 29,6 |
| 109 | High Angle | Gram-positive cocci | 10 | 13 | 7 | Absent | Absent | 40,17 |
| 110 | Norm Angle | Gram-positive cocci | 15 | 18 | 7 | Absent | Occasional | 29,7 |
| 111 | Norm Angle | Gram-positive bacilli | 8 | 9 | 7 | Absent | Absent | 34,2 |
| 112 | Norm Angle | Gram-positive bacilli | 15 | 9 | 7 | Absent | Absent | 29,4 |
| 113 | Norm Angle | Gram-positive bacilli | 14 | 5 | 7 | Absent | Absent | 27,4 |
| 114 | High Angle | Gram-positive bacilli | 11 | 21 | 7 | Absent | Absent | 36,64 |
| 115 | Norm Angle | Gram-positive bacilli | 11 | 15 | 7,5 | Absent | Absent | 27,6 |
| 116 | High Angle | Gram-positive bacilli | 15 | 15 | 7,5 | Absent | Absent | 38 |
| 117 | Norm Angle | Gram-positive bacilli | 12 | 5 | 7,5 | Absent | Absent | 34,5 |
| 118 | High Angle | Gram-positive bacilli | 17 | 1 | 7 | Absent | Absent | 37,06 |
| 119 | High Angle | Gram-positive cocci and bacilli | 13 | 10 | 7,5 | Absent | Absent | 39,6 |
| 120 | High Angle | C. Albicans | 15 | 12 | 7,5 | Absent | Continuous | 45,9 |
| 121 | High Angle | Gram-positive bacilli | 13 | 17 | 7,5 | Absent | Absent | 36,9 |
| 122 | High Angle | Gram-positive cocci | 17 | 23 | 7,5 | Continuous | Continuous | 58,31 |
| 123 | High Angle | Bakteri | 14 | 2 | 7,5 | Absent | Absent | 35,02 |
| 124 | High Angle | Gram-positive cocci and bacilli | 14 | 10 | 7,5 | Absent | Absent | 36,7 |
| 125 | High Angle | Candida Spp | 14 | 12 | 7 | Absent | Absent | 39,6 |
| 126 | High Angle | Absent | 15 | 13 | 7,5 | Absent | Absent | 38,91 |
| 127 | High Angle | Absent | 11 | 18 | 7,5 | Absent | Absent | 38,6 |
| 128 | Norm Angle | Gram-positive bacilli | 12 | 4 | 7 | Absent | Absent | 34,46 |
| 129 | High Angle | Gram-positive bacilli | 9 | 24 | 7 | Absent | Continuous | 38,67 |
| 130 | High Angle | Gram-positive cocci and bacilli | 15 | 15 | 8 | Absent | Continuous | 42,64 |
| 131 | High Angle | Gram-positive cocci | 13 | 11 | 7,5 | Absent | Continuous | 43,02 |
| 132 | Norm Angle | C. Albicans | 17 | 5 | 7 | Absent | Occasional | 35,6 |
| 133 | High Angle | Gram-positive cocci | 16 | 3 | 7,5 | Absent | Absent | 38,5 |
| 134 | High Angle | C. Albicans | 12 | 13 | 7 | Absent | Continuous | 46,44 |
| 135 | High Angle | Gram-positive cocci | 17 | 5 | 7 | Absent | Absent | 37,6 |
| 136 | High Angle | Gram-positive cocci | 16 | 3 | 7,5 | Absent | Absent | 37,15 |
| 137 | High Angle | Gram-positive cocci | 17 | 10 | 7 | Absent | Absent | 36,09 |
| 138 | High Angle | Gram-positive cocci | 12 | 14 | 7,5 | Continuous | Absent | 46,26 |
| 139 | High Angle | C. Dubliniensis | 13 | 10 | 7 | Absent | Absent | 38,96 |
| 140 | High Angle | Gram-positive cocci | 17 | 5 | 7 | Absent | Absent | 38,58 |
| 141 | High Angle | Candida Spp | 14 | 8 | 7,5 | Continuous | Absent | 40,74 |
| 142 | Norm Angle | Gram-positive cocci | 14 | 7 | 7,5 | Absent | Absent | 35,2 |
| 143 | High Angle | Gram-positive cocci | 17 | 9 | 7,50 | Absent | Continuous | 45,35 |
| 144 | High Angle | C. Dubliniensis | 14 | 0.5 | 6,5 | Continuous | Continuous | 42,24 |
| 145 | High Angle | Gram-positive cocci | 15 | 5 | 7 | Absent | Absent | 37,99 |
| 146 | High Angle | Absent | 15 | 15 | 8 | Absent | Continuous | 36,7 |
| 147 | High Angle | C. Guilliermondii | 13 | 10 | 7,50 | Absent | Absent | 37,15 |
| 148 | High Angle | Absent | 15 | 3 | 8 | Absent | Absent | 38,27 |
| 149 | High Angle | Gram-positive cocci | 16 | 9 | 7 | Absent | Absent | 37,12 |
| 150 | High Angle | Gram-positive cocci | 17 | 5 | 7 | Absent | Absent | 37,21 |
